# Supplementary material for: Mesenchymal-Stem-Cell-Derived Extracellular Vesicles Attenuate Brain Injury in Escherichia coli Meningitis in Newborn Rats
Source: Life (Basel). 2022 Jul 11;12(7):1030. doi: 10.3390/life12071030 (PMC9319453; doi:10.3390/life12071030)
Supplement: Supplementary file 1 [file life-12-01030-s001.zip › life-1762413-supplementary.pdf]

Supplementary Materials

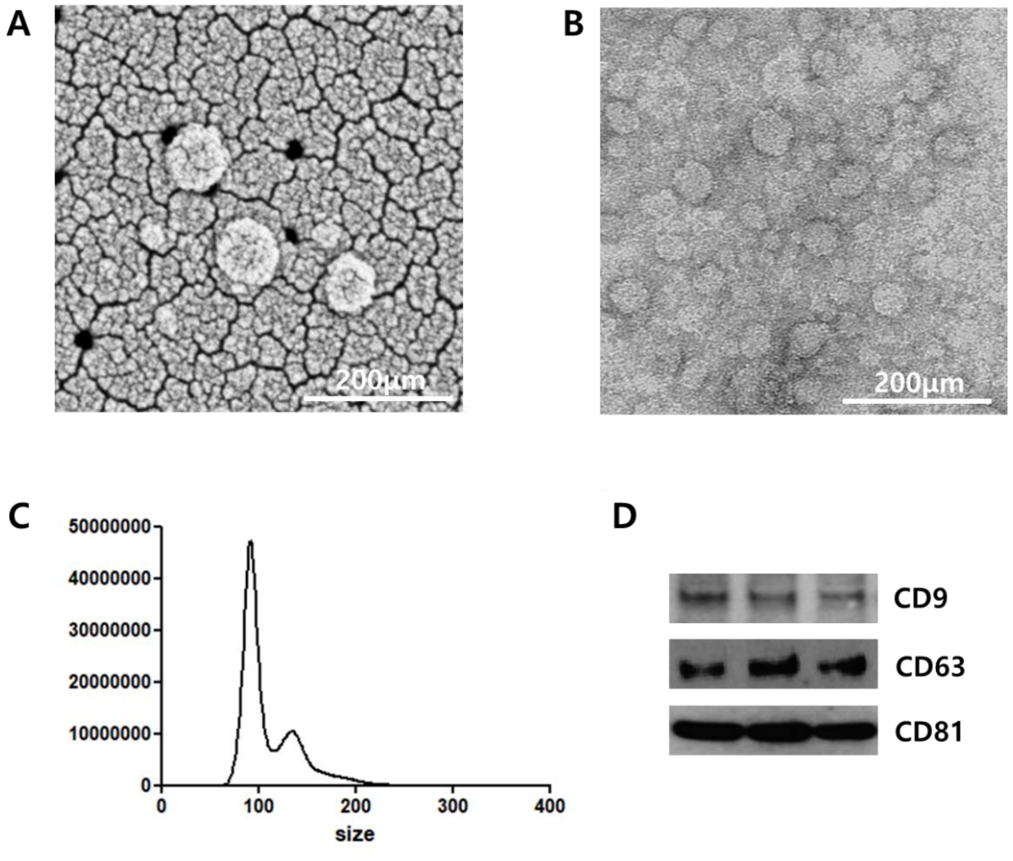

Supplementary Figure S1. Characterization of extracellular vesicles (EVs). (A) Scanning electron microscopic image of EVs loaded on a polycarbonate membrane. EVs were isolated from conditioned media of cultures of human Wharton's jelly-derived mesenchymal stem cells using ultra-centrifugation. (B) Transmission electron microscope photograph of EVs. (C) Particle size distribution of EVs using Nanoparticle Tracking Analysis software. The Y-axis represents the number of EVs and the X-axis represents the size of EVs. (D) Western blots for three consecutively separated EVs, indicating exosome marker proteins of CD63, CD9, and CD81, respectively.

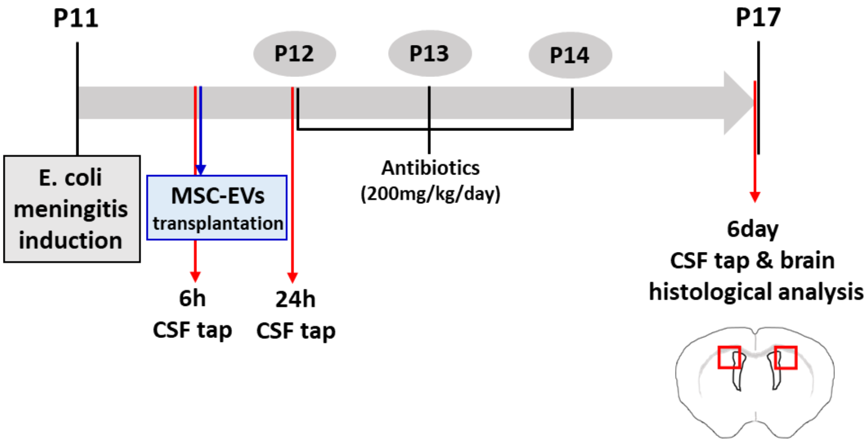

Supplementary Figure S2. Experimental protocol. All injections were performed directly into the lateral ventricle. 5×10^2 CFU of *E. coli* were inoculated into the left ventricle of P11 rat pups, and MSC-EVs isolated from 1×10^5 MSC were transplanted into the right ventricle 24 hours later. CSF was tapped three times; immediately before 1) transplantation of MSC-EVs at 6 h, 2) first administration of antibiotics at 24 h, and 3) sacrifice at 6 days after animal modeling. Histological analyses of the brain tissues were performed in the periventricular area marked with red squares. P; postnatal day, CSF; cerebrospinal fluid, MSC-EVs; EVs isolated from mesenchymal stem cells

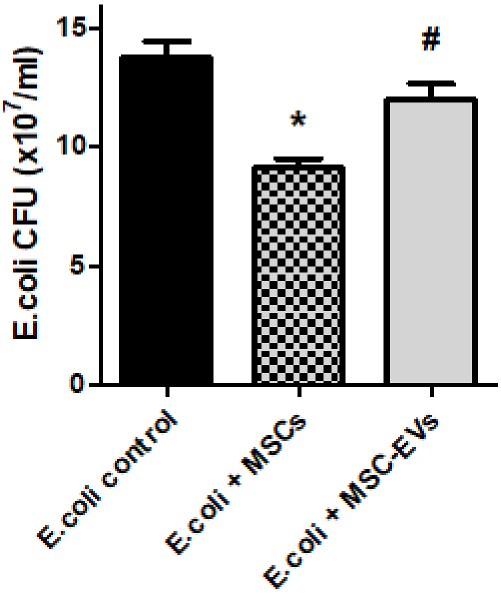

Supplementary Figure S3. Treatment of MSCs (1×10^5/ml), but not MSC-EVs (10μg/ml), significantly inhibited bacterial growth in culture media. Data are expressed as mean ± SEM. * $p < 0.05$ vs. *E. coli* control, # $p < 0.05$ vs. *E. coli* cultured with MSCs
